# Viable appendico-ileal knotting resulting in acute small bowel obstruction: a case report from Ethiopia

**DOI:** 10.1093/jscr/rjaf1109

**Published:** 2026-01-21

**Authors:** Amanuel Mesfin Oljira, Abebe Megersa Tebelu, Motume Birhanu Abdisa

**Affiliations:** Department of Surgery, Ambo University College of Medicine and Health Science, Addis Ababa–Nekemte Road, PO Box 19, Ambo, West Shewa Zone, Oromia Regional State, Ethiopia; Department of Surgery, Ambo University College of Medicine and Health Science, Addis Ababa–Nekemte Road, PO Box 19, Ambo, West Shewa Zone, Oromia Regional State, Ethiopia; Department of Surgery, Ambo University College of Medicine and Health Science, Addis Ababa–Nekemte Road, PO Box 19, Ambo, West Shewa Zone, Oromia Regional State, Ethiopia

**Keywords:** appendico-ileal knotting, viable bowel, closed-loop obstruction, small bowel obstruction, appendectomy; Ethiopia

## Abstract

Appendico-ileal knotting is a rare cause of small bowel obstruction, usually complicated by strangulation and resection of bowel. A 32-year-old woman presented with 24-hour abdominal pain, bilious vomiting, distension, and obstipation. Visible peristalsis was noted on physical examination; a radiograph showed multiple air–fluid levels and a dilated small bowel. Emergency laparotomy revealed an inflamed appendix, which formed a constricting ring around a loop of 15 cm of distal ileum, which was viable. The knot was released with retrograde appendectomy without bowel resection. A follow-up 1 year later revealed no complications and a complete recovery. Early recognition and appropriate timely intervention can help in the preservation of bowel viability in appendico-ileal knotting, and hence organ-sparing management can be achieved.

## Introduction

Small bowel obstruction (SBO) is a common surgical emergency. Most cases result from adhesions, hernias, or malignancy [1]; rare mechanical etiologies such as intestinal knotting (ileo-sigmoid, ileo-ileal, and appendico-ileal knotting, AIK) account for <1% [2, 3]. In AIK, the appendix forms a constricting ring around the ileum, creating a closed loop with a high risk of rapid ischemia and gangrene [4, 5]. Since first described in 1901, AIK has been reported sporadically across Africa, Asia, and elsewhere, often with delayed presentation and frequent bowel resection [6–9]. Diagnostic delay and morbidity remain common, including reports from Nigeria [5], India [6, 10], and Ethiopia [7, 11]. Preoperative identification is difficult; computed tomography (CT) can suggest a closed loop or appendiceal tie, but diagnosis is usually intraoperative [10]. This case is notable for viable AIK successfully treated with appendectomy alone, in contrast to the predominance of gangrenous cases requiring resection [5–7, 10–13]. It highlights that early presentation and prompt surgery can preserve bowel viability and challenges the assumption that AIK invariably progresses to strangulation.

## Case report

A 32-year-old woman with no prior abdominal surgery presented after 24 hours of severe crampy abdominal pain, frequent bilious vomiting, progressive distension, and constipation. She had no major comorbidities. Her vitals were as follows: heart rate (HR) 110 bpm, blood pressure (BP) 100/70 mmHg, respiratory rate (RR) 20/minute, temperature 36.5°C, and SpO₂ 96%. The abdomen was distended with visible peristalsis, hyperactive bowel sounds, and mild direct generalized tenderness; a rectal exam revealed stool. There were no inguinal, femoral, or umbilical hernias and no peritonism (Table 1).

**Table 1:** Clinical timeline

Date/Interval	Event
Day 0	Onset of severe abdominal pain, bilious vomiting, distension, and obstipation
Day 1	Emergency department presentation; resuscitation; erect abdominal radiograph showing multiple central air–fluid levels
Same day	Exploratory laparotomy performed; appendico-ileal knotting identified; knot released via retrograde appendectomy
Postoperative Day 7	Patient discharged in stable condition
10 days post-op	First follow-up: no complications
1 month post-op	Second follow-up: normal recovery
3 months post-op	Third follow-up: no recurrence
6 months post-op	Fourth follow-up: asymptomatic
12 months post-op	Final follow-up: complete recovery, no bowel dysfunction

**Figure 1:**
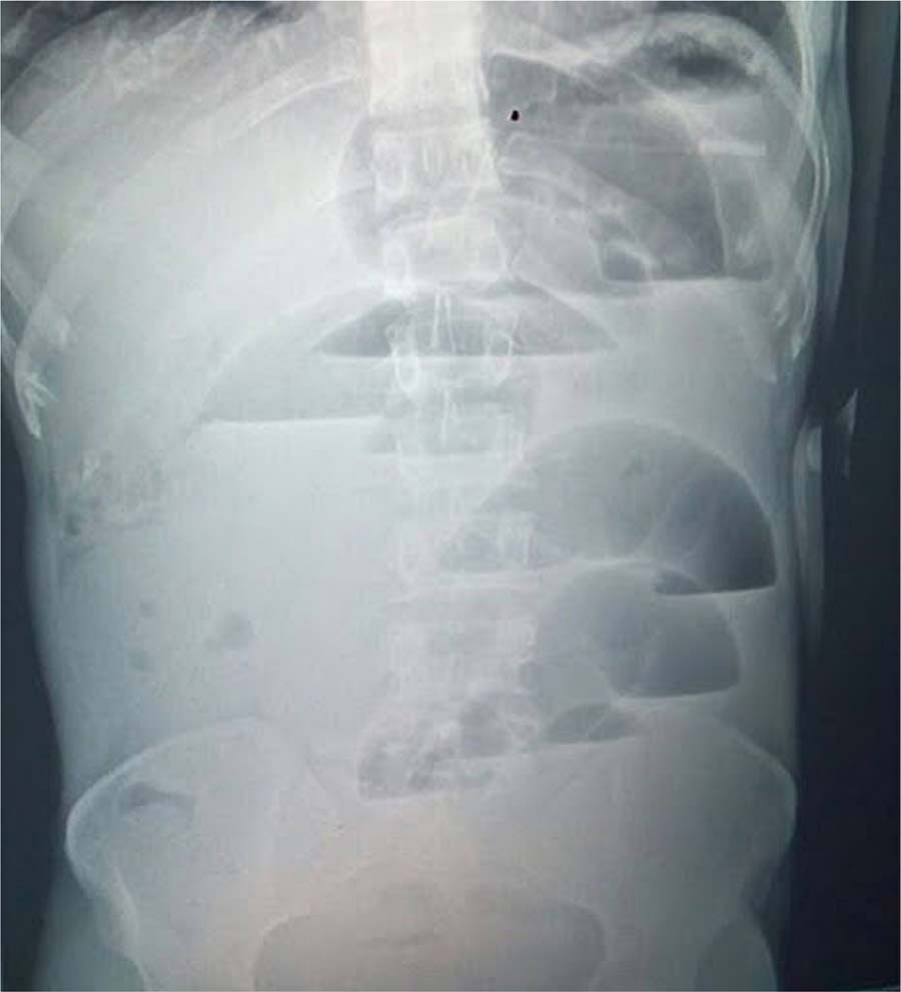
Erect abdominal radiograph showing multiple air–fluid levels and dilated bowel loops.

The laboratory findings included hemoglobin at 13.2 g/dl, a white blood cell (WBC) count of 9.4 × 10^9^/l (78% neutrophils), platelets at 256 × 10^9^/l; sodium at 138 mmol/l, potassium at 3.1 mmol/l, chloride at 102 mmol/l, bicarbonate at 24 mmol/l, creatinine at 0.8 mg/dl, and lactate at 1.2 mmol/l; urine pregnancy test was negative. Erect abdominal radiograph (24 hours from symptom onset) showed multiple central air–fluid levels and dilated small bowel loops (Fig. 1). Without prior surgery, adhesive obstruction was unlikely. The differential diagnoses included internal hernia, strangulated external hernia, ileo-sigmoid knotting, and rarer causes such as appendicular ileo-sigmoid knotting (AIK).

**Figure 2:**
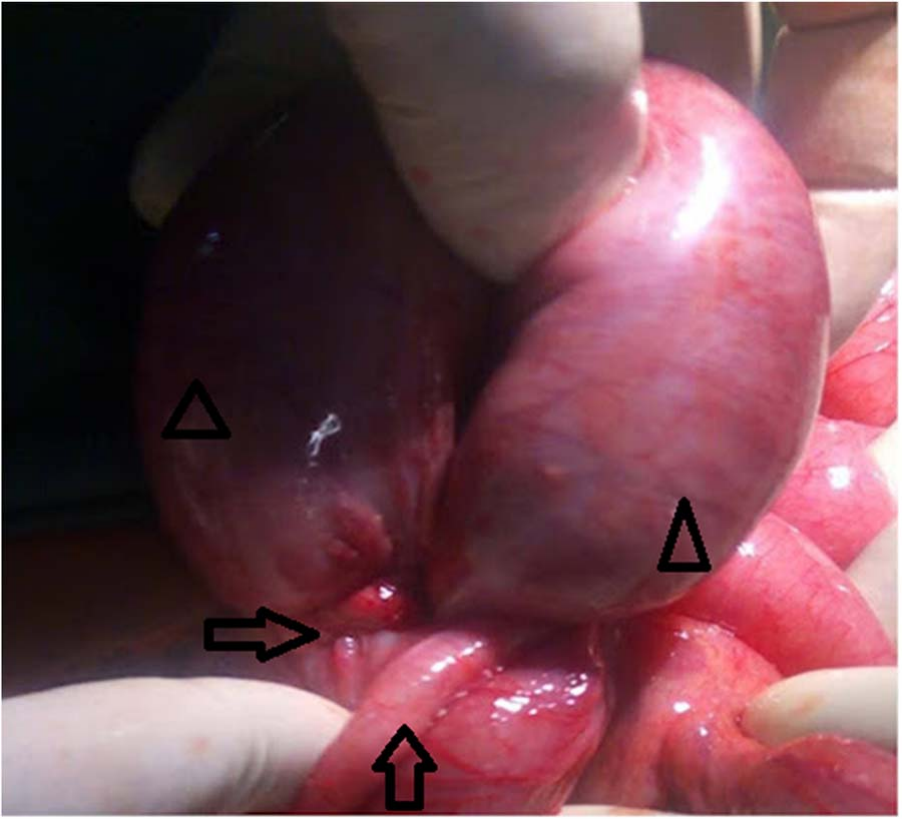
Inflamed appendix forming a constricting ring (arrow) around a loop of viable distal ileum (arrow head).

Initial management comprised isotonic IV fluids, nasogastric decompression, and potassium replacement (20 mEq KCl in 1 l normal saline over 3 hours, then 20 mEq over 3 hours), and potassium was normalized to 3.8 mmol/l preoperatively. Despite this, pain and bilious vomiting persisted, suggesting failed conservative therapy and a closed loop at risk for ischemia. CT was unavailable; given clinical deterioration, urgent laparotomy was performed, confirming AIK intraoperatively (Fig. 2).

**Figure 3:**
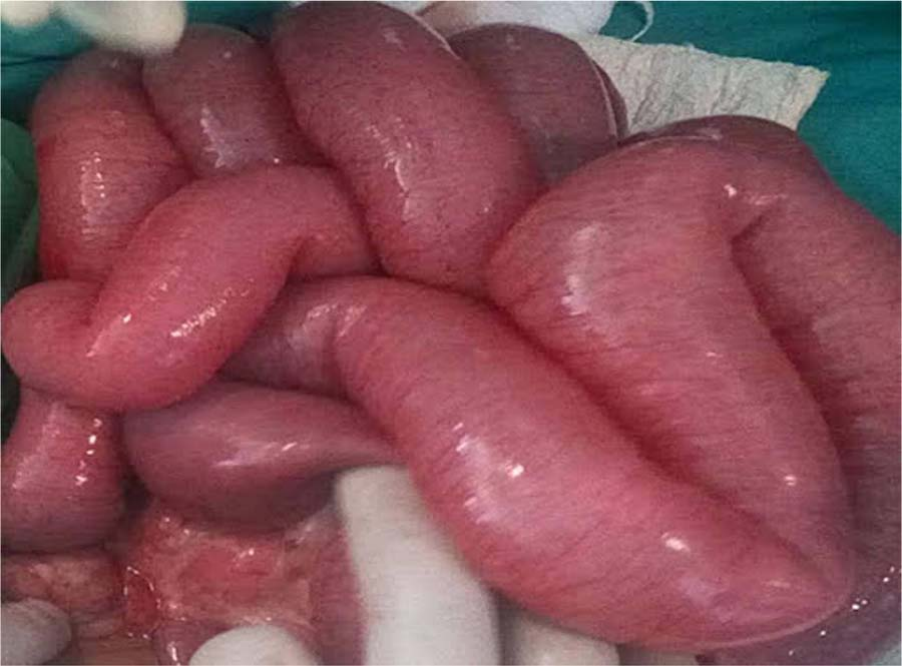
Viable distended proximal small bowel loops.

After consent, prophylactic ceftriaxone 1 g IV and metronidazole 500 mg IV were administered 30 minutes preincision and continued for 24 hours, per clean-contaminated prophylaxis. Midline laparotomy revealed minimal reactive fluid and an inflamed appendix forming a tight ring around ~15 cm of distal ileum, <10 cm proximal to the ileocecal valve (Fig. 2). The appendiceal tip adhered to the distal ileal mesentery, causing luminal obstruction without a complete tourniquet. There was no mesenteric twist or pedicle occlusion; mesenteric arterial pulsations were palpable with brisk capillary refill and active peristalsis. The proximal bowel was markedly distended yet viable (Fig. 3).

The knot was released by gentle traction and division of the appendix at its base (retrograde appendectomy), with mesenteric vessels protected; no bowel decompression was performed. Viability—uniform pink color, active peristalsis, palpable mesenteric pulsations—remained satisfactory after release. Objective perfusion adjuncts (Doppler, indocyanine-green angiography) were unavailable.

Postoperatively, the patient received 24 hours of IV antibiotics, early ambulation, and graded diet advancement; bowel sounds returned on postoperative Day 2. She was discharged on Day 7 with uneventful recovery and planned follow-up at 10 days, 1 month, 3 months, 6 months, and 1 year (Table 1). Histopathology of the appendix was not performed due to lack of pathology services.

## Discussion

AIK is a rare cause of mechanical SBO and often progresses rapidly to strangulation and gangrene, necessitating resection [5–7, 10–12]. Many African and South Asian reports document delayed presentation and extensive resections, including Nigerian pediatric cases with volvulus and strangulation [5] and Indian series requiring hemicolectomy [6, 10]; similar high morbidity has been reported in Pakistan and Ethiopia when diagnosis is delayed [7, 11].

In contrast, our patient presented within 24 hours; immediate exploration confirmed a viable ileal segment, enabling appendectomy without resection. This aligns with a limited body of evidence indicating that timely intervention and systematic assessment of viability (color, peristalsis, mesenteric pulsation) can avert unnecessary bowel loss [1–4]. Organ-sparing outcomes have likewise been described in appendicular band syndromes and stump-related obstructions in high-resource settings [8, 9, 13].

Preoperative diagnosis is challenging; while CT may show a closed loop or appendicular ring, most diagnoses—including ours—are made intraoperatively [10]. In centers with CT, features prompting early operative management include U/C-shaped closed loops, adjacent transition points, beak sign, converging mesentery, venous congestion, and—when present—the mesenteric whirl sign; a right-lower-quadrant transition with mesenteric crowding should raise suspicion for appendiceal banding.

Pathophysiologically, our intraoperative findings suggest a partial ring effect with preserved arterial inflow and adequate venous return, preventing the venous congestion, transmural edema, and ischemia typical of closed-loop obstruction. Absence of mesenteric twist likely minimized shear to the vasa recta and forestalled rapid strangulation.

The limitations include the lack of preoperative CT, single-case generalizability, and reliance on clinical viability criteria without objective perfusion adjuncts.

This case demonstrates that appendico-ileal knotting does not invariably result in ischemia. Early surgical exploration and careful intraoperative assessment of viability can permit organ-sparing management with appendectomy alone when the involved ileum remains viable [2, 13, 14].

## Patient perspective

“I was frightened of the sudden pain and vomiting. After surgery, I felt better, and I was happy that no intestines were removed.” Thank you for the quick care and recovery.
